# Maine Veterinary Medical Association

**Published:** 1896-03

**Authors:** W. L. West

**Affiliations:** Secretary


Secretary.

### MAINE VETERINARY MEDICAL ASSOCIATION.

THE semi-annual meeting of the Maine Veterinary Medical Association was held at the Greble House, Portland. The meeting was called to order, with President Bailey in the chair. Drs. Lord, Huntington, Choate, Russell, Perry, Purcell, Joly, Long, and West answered to the roll-call. The minutes of the previous meeting were read and approved. Dr. P. L. Stevens, of Farmington, was unanimously elected to membership. The following officers were unanimously elected: President, Dr. F. L. Russell, Orono; Vice-President, Dr. J. W. Huntington, Portland; Secretary, Dr. W. L. West, Ellsworth; Treasurer, Dr. A. Joly, Waterville.

Dr. West read a paper on "Hemorrhagic Enteritis," which was freely discussed. Dr. Bailey then made some remarks anent the tuberculin test, which called forth a spirited discussion and resulted in a vote by the Association that: 1st. The tuberculin used by members shall be that prepared by Koch and known as Koch's tuberculin. 2d. The minimum price for testing cattle shall be: For 20 or less, $1.50 per head; for 20 to 30, $1.25 per head; for 30 or more, $1.00 per head.

The meeting then adjourned to meet at Lewiston, July 15th next.

W. L. WEST, V.S.,
Secretary.

